# Cognitive functioning in adult psychiatric patients with and without attention‐deficit/hyperactivity disorder

**DOI:** 10.1002/brb3.3626

**Published:** 2024-07-25

**Authors:** D. Eberhard, C. Gillberg, E. Billstedt

**Affiliations:** ^1^ Gillberg Neuropsychiatry Centre, Sahlgrenska Academy, University of Gothenburg Gothenburg Sweden; ^2^ Child Neuropsychiatric Clinic Queen Silvia Children´s Hospital Gothenburg Sweden

**Keywords:** ADHD, adult, cognition, intellectual function, wais

## Abstract

**Introduction:**

Studies of cognitive functioning in patients with attention‐deficit/hyperactivity disorder (ADHD) have often used healthy comparison groups. The present study examines cognitive profiles, including general intellectual and executive functions, in a young adult psychiatric outpatient clientele with ADHD and evaluates whether their cognitive profiles can help differentiate them from patients with non‐ADHD‐associated psychiatric disorders.

**Methods:**

The study group comprised 141 young adult psychiatric patients (age range 18–25 years) of whom 78 had ADHD. Comprehensive neuropsychological assessment included the Wechsler Adult Intelligence Scale, 4th version and subtests from Delis–Kaplan Executive Function System. Clinical psychiatric assessments and diagnostic evaluation were performed.

**Results:**

The ADHD group (including all subtypes) had significantly lower verbal comprehension and full‐scale intelligence quotient than the non‐ADHD group. Tests measuring working memory or executive function did not separate those with and without ADHD.

**Conclusion:**

The results of our study suggest that, except for the need to establish overall cognitive performance level, the clinical implication of testing is small if the purpose is to “rule out” an ADHD diagnosis.

## 1 INTRODUCTION

In psychiatry, the focus has traditionally been on “emotions” and cognitive aspects, by and large, have gained less attention. Given the close relationship between emotions and cognition, and the negative impact on overall functioning of cognitive deficits, this seems to have been irrational (Millan et al., 2012). Cognitive deficits, including executive dysfunction, are closely associated with adaptive functions (Harrison & Oakland, 2008; Nyrenius & Billstedt, 2020) and with quality of life (Knight et al., 2020). Adaptive behavior is defined as everyday skills needed to function independently and meeting the demands of one's community (Tassé, 2021). Executive function is a complex construct, which involves the organization and direction of cognition, emotions, and behaviors (Strong et al., 2010). The most often described executive functions are inhibition (including behavioral inhibition and interference control), working memory, and cognitive flexibility (e.g., Diamond, 2013; Karr et al., 2018). Even though cognitive functions, and the measurement of cognition, are modified by several factors (e.g., education, motivation) specific patterns of cognitive functioning in different psychiatric conditions have been described (e.g., O'Sullivan & Newman, 2014; Kriesche et al., 2022; Marinopoulou et al., 2016)

Attention‐deficit/hyperactivity disorder (ADHD), which is the most prevalent of the neurodevelopmental disorders, has been described to be associated with cognitive deficits (Gillberg, 2021). A large meta‐analysis including cases of all ages with ADHD, reported that it was associated with moderately lower IQ (Frazier et al., 2004). Full‐scale intelligence quotient (FSIQ) was significantly lower in ADHD groups compared to healthy controls with effect size weighted *d *= 0.61, which corresponds to a nine‐point difference in FSIQ (Frazier et al., 2004). However, another meta‐analysis that only included adults with ADHD reported only small IQ deficits, which were described as not being clinically meaningful (Bridgett & Walker, 2006). The literature is more unambiguous when it comes to the association between ADHD and executive dysfunction. A large meta‐analysis (Schoechlin & Engel, 2005) found ADHD to be associated with small to moderate difficulties with working memory, abstract problem solving, focused attention, and sustained attention. Another meta‐analysis supported this association but also found that the working memory deficits got more pronounced with age (Ramos et al., 2020). A more recent meta‐analysis (Onandia‐Hinchado et al., 2021) confirmed findings from the Schoechlin and Engel study, that ADHD is associated with difficulties related to sustained attention, processing speed, and working memory. The authors also reported an association between ADHD and reduced inhibition, particularly with emphasis on reward delay and interference control or the ability to resist distracting stimuli. In a very large study of >1500 children and adolescents, executive dysfunction was found to be specific to the inattention domain of ADHD (Sabhlok et al., 2022).

The estimated prevalence of ADHD in adulthood is about 4% (Kessler et al., 2006), however, a significant proportion of those with the disorder are not identified with ADHD due to comorbid psychiatric conditions overshadowing the ADHD symptoms (Culpepper & Mattingly, 2010; Ginsberg et al., 2014; Newcorn et al., 2007). This would indicate that adults with ADHD might often be referred to a psychiatric clinic for assessment and treatment with the focus on other psychiatric disorders and not their ADHD.

Studies relating to cognitive functioning in adults with ADHD have often used “healthy controls” and have concluded that ADHD in adulthood is associated with deficits in several cognitive domains (Onandia‐Hinchado et al. 2021). Studies of cognitive deficits in adult ADHD have included predominately males (Schoechlin & Engel, 2005). This means that there is a need for studies of cognitive function in adults (males and females) with ADHD who have not been diagnosed in childhood, but in whom other psychiatric problems have prompted psychiatric assessment and to include clinical cases without ADHD as a comparison group.

The aim of the present study was to examine the cognitive profiles including general intellectual and executive functions in a young adult psychiatric outpatient clientele and to evaluate whether or not the cognitive profiles could help differentiate ADHD from non‐ADHD‐associated psychiatric disorders.

## 2 MATERIALS AND METHODS

### 2.1 Procedure

All 18‐ to 25‐year‐old new patients (*n* = 172) attending an adult outpatient psychiatric (AOP) clinic between November 2015 and January 2018 (with a break between March and August 2016) were invited to take part in the current study. Patients with a preliminary or already established diagnosis of ADHD as well as those who primarily did not seek help for ADHD were invited to take part in the clinical research assessment including a cognitive evaluation. In real clinical practice the focus of assessment is often the referral question, whereas in the current study, all participants underwent a broad psychiatric assessment. It was mandatory for those who accepted participation in the study to undergo in‐depth clinical psychiatric assessment; cognitive assessment and completing questionnaires were voluntary. The patients at the clinic were considered representative of young people seeking help at AOP clinics in Stockholm except for patients with psychotic disorder (of whom the majority had been referred directly to a specialized psychosis unit).

#### 2.1.1 Clinical examination

The study group was extensively assessed regarding psychiatric problems, neurodevelopmental disorders (NDD), and cognitive functioning. The *Mini International Neuropsychiatric Interview* (M.I.N.I.) (Sheehan et al., 1998) was used as a structured broad psychiatric interview for psychiatric diagnosis according to the Diagnostic and Statistical Manual of Mental Disorders (DSM) and in cases with suspected personality disorder, the *Structured Clinical Interview guide for Diagnosis of Axis II disorders* (SCID‐II, First et al., 1997) was used.

The assessments were performed by the same two clinically experienced psychiatrists, two clinically experienced psychologists, and one nurse, except in two cases where there was a third psychiatrist doing the assessments. The psychiatrist in the team also made a physical evaluation of each participant. Final clinical diagnoses, performed by the psychiatrist, were based on all available information provided at the clinical psychiatric interview, self‐rating questionnaires, and the clinical impression of the respondent according to the DSM‐IV‐TR (APA, 2000) and DSM‐5 criteria (APA, 2013).

#### 2.1.2 Self‐rating questionnaire

The *Wender Utah Rating Scale‐25* (WURS‐25) (Ward et al., 1993) and the *ADHD‐Rating Scale* (ADHD‐RS) (DuPaul et al., 1998) were used in the study. WURS is an adult questionnaire with ADHD symptoms in childhood, whereas ADHD‐RS, on the other hand, is a rating scale of current ADHD symptoms. The *Autism Symptom SElf‐ReporT for adolescents and adults* (ASSERT) (Posserud et al., 2013) and the *Adult Autism Spectrum Quotient* (AQ) (Baron‐Cohen et al., 2006) were used for ASD screening. The *Alcohol Use Disorders Identification Test* (Saunders et al., 1993), the *Drug Use Disorders Identification* (Berman et al., 2005), and the *Fagerström Test for Nicotine Dependence* (Heatherton et al., 1991). The results from these questionnaires are presented in Eberhard et al. (2022).

Neuropsychological assessments were performed by clinically licensed psychologists, and the psychiatric clinical evaluation was performed by an experienced psychiatrist. The clinical diagnosis was based on the information, provided at the clinical psychiatric interview, self‐rating questionnaires, and the clinical impression of the respondent according to the text revision of the fourth edition of the *Diagnostic and statistical manual of mental disorders* (DSM‐IV‐TR) (APA, 2000) and also in accordance with DSM‐5 criteria (APA, 2013). The psychiatrist involved in the diagnostic decision also evaluated symptom severity using the Clinical Global Impression Scale (CGI) (Guy, 1976), a seven‐point scale on which higher scores indicate more severe problems.

The study participants were invited to complete self‐rating questionnaires and parent questionnaires were sent to the patient´s parent if the participant permitted this. For measuring ADHD symptoms, we used the Wender Utah Rating Scale‐25 item self‐rating questionnaire (WURS‐25) (Ward et al., 1993), which is an adult scale with questions relating to ADHD symptoms in childhood. WURS‐25 provides a total sum score (range 0–100). Internal consistency of the WURS has been reported at 0.94 (Kouros et al., 2018). The parent questionnaire in the study was the Five to Fifteen questionnaire (FTF) (Kadesjö et al., 2004). The FTF is used to assess development and behavior in children aged 5–15 years but it has been used in retrospective studies of young people above 15 years of age (Lugnegård & Bejerot, 2019).

### 2.2 Cognitive instruments

The Wechsler Adult Intelligence Scale‐Fourth Edition (WAIS‐IV) (Wechsler, 2008) was used to provide a measure of general intellectual functioning (FSIQ), and four index scores, Verbal Comprehension Index (VCI), Perceptual Reasoning Index (PRI), Working Memory Index (WMI), and Processing Speed Index (PSI).

The Delis‐Kaplan Executive Function System (D‐KEFS) (Delis et at. 2001) is an inventory including generic versions of different tests aimed at assessing a variety of executive functions for ages 8–89. The D‐KEFS provides US norms on over 1500 individuals. It was administered to 136 participants in the study. The total achievement scores from the D‐KEFS tests were used in the analyses since they reflect global achievement on these tests. The results are presented in scaled scores, with a mean of 10 and a standard deviation of 3. The following D‐KEFS tests were used in this study:

Trail Making Test (TMT) assesses cognitive flexibility, attention, and resistance to distraction. TMT involves five conditions: Visual scanning, number sequencing, letter sequencing, number‐letter switching, and motor speed. The main score is the completion time of the different conditions. The TMT condition most demanding of executive functioning is the number‐letter switching condition when the participant switches back and forth between connecting numbers and letters (i.e., 1, A, 2, B, and so on).

Verbal Fluency Test requires lexical production and automatic lexical access and reflects efficient lexical organization. Verbal Fluency Test comprises three subtests of which all require verbal response within 1 min. Letter fluency requires generation of words that start with a specific letter. Category fluency requires generation of words that belong to a specific semantic category (e.g., animals), and Category switching requires the examinee to alternate, within 1 min, between two different semantic categories (fruits and furniture). All three subtests are considered to measure aspects of executive functioning.

Color–Word Interference Test (CWIT) requires selective or focused attention, the ability to shift from one perceptual set to another, and the ability to inhibit inappropriate responding. The participant is presented with a page that contains a series of red, green, and blue squares or color names. CWIT consists of four subtests: color naming (to quickly say the names of the colors), color word reading (to quickly read the color names), inhibition (the participant has to say the color of the word that is printed incongruently in different colors), and inhibition/switching (the participant says the ink color but to read the word out loud when the color names are enclosed within boxes). Performance is measured by completion time on each of the four trials. Inhibition and inhibition/switching are considered being most sensitive of executive impairment.

In the Twenty Questions Test, which requires strategic thinking, is stimulus page presented containing 30 common objects. The participant has to identify an unknown target object using the fewest number of yes/no questions. The test yields an abstraction score which represents participants’ ability to, with the first question, eliminate the most objects.

### 2.3 Participants

Of 172 eligible patients, 170 accepted (107 women and 63 men), participation in the original clinical study (Eberhard et al., 2022). Note that 141 (82%) of these participated in the cognitive assessment, 90 women and 51 men, who together constitute the current cognitive study group. One male, with no ADHD diagnosis, did not take part in the WAIS test but participated in the other cognitive tests meaning that WAIS was administered to 140 participants. The mean age of those participating in cognitive assessment was 20.8 years (SD = 2.3) compared to 21 years (SD = 2.6) in the group who did not participate in cognitive assessment. A chi‐square test for independence (with Yates Continuity Correction) indicated no significant association between having a diagnosis of ADHD and participation/ nonparticipation, *χ* ^2^ (1, *n *= 167) = .098, *p *= .225, and *φ *= .098.

In the cognitive study group, 78 (55%) were students, 41 (29%) were employed, and the remaining 22 (16%) were unemployed. The highest achieved level of education in the study group was lower secondary school (*n* = 6, 4%), upper secondary school but not fully completed (*n* = 70, 50%), completed upper secondary school (*n* = 38, 27%) or post‐secondary school (*n* = 27, 19%). A majority of the study group still lived with a parent (*n* = 84, 60%). However, 51 participants (36%) lived on their own, or in another living condition such as group home or other similar living conditions (*n* = 6, 4%).

#### 2.3.1 ADHD group and non‐ADHD group

We divided the 141 participants in the cognitive study group into (a) ADHD group (*n* = 78, 55%) and (b) non‐ADHD group (*n* = 63, 45%) based on the diagnostic procedure described above leading to ADHD diagnosis according to DSM‐5 criteria. Twenty seven (35%) in the ADHD group had ADHD inattentive subtype and the remaining 51 (65%) had ADHD combined type. See Table 1 for psychiatric diagnoses and demographic data.

**TABLE 1:** Demographic and psychiatric descriptive of the attention‐deficit/hyperactivity disorder (ADHD) group and non‐ADHD group.

	Non‐ADHD group, *n* = 63 N (%)	ADHD group, *n* = 78
Mean age (SD)	20.9 (2.3)	20.7 (2.4)
Females N (%)	44 (70)	46 (59)
Highest achieved education		
Lower secondary school	2 (3)	4 (5)
Uncomplete secondary school	25 (40)	45 (58)
Upper secondary school	19 (30)	19 (24)
Post secondary school	17 (27)	10 (13)
Occupation		
Student	31 (49)	47 (60)
Employment	18 (29)	23 (30)
Unemployed	14 (22)	8 (10)
Psychiatric diagnoses		
Autism spectrum disorder	15 (24)	12 (15)
Affective disorder	24 (40)	2 (3)
Anxiety disorder	23 (38)	9 (12)
Personality disorder	7 (11)	17 (22)
Eating disorder	8 (13)	2 (3)
Drug/alcohol abuse	8 (13)	10 (13)
CGI, M (SD)	3.8 (.65)	3.8 (.68)
WURS, M (SD)	32.1 (16.1)	46.6 (19.6)

Abbreviations: CGI, clinical global impression scale; WURS, Wender Utah Rating Scale‐25 item scale.

No differences regarding age, proportion of females/males, or CGI scores were found between the ADHD group and the non‐ADHD group. However, the ADHD group scored significantly higher on the WURS‐25 (M = 46.6, SD = 19.9) than the non‐ADHD group (M = 32.1, SD = 16.1, *t*(128) = −4.530, and *p *< .001)

### 2.4 Statistical analysis

Student's *t*‐test was used to compare WAIS results in the ADHD and non‐ADHD subgroups. Logistic regression analysis was used to explore the impact of the independent variables (significant variables from D‐KEFS and WAIS indices) on the dependent variable (ADHD, no ADHD). One‐way analysis of variance was used to compare means between three groups (non‐ADHD, ADHD‐combined type, and ADHD‐inattentive type). The homogeneity of variance assumption was not violated according to Levene´s test for homogeneity of variance. One‐sample *T*‐test was used to compare D‐KEFS with the norms.

#### 2.4.1 Ethics

The study was approved by the Research Ethics Committee at the University of Gothenburg (No 047‐14). Written informed consent was obtained from all participants. It was clearly stated in the letter of consent that there would be no negative consequences if patients declined to take part in the study. No economical compensation was given.

## 3 RESULTS

### 3.1 WAIS tests results

The FSIQ and the index results in the total study group were in the average range (Table 2). The only gender difference was found for WMI where females had lower scores than males (males: M = 101.6, SD = 14.2; females: M = 93.9, SD = 13.3, *t*(138) = 3.196, and *p *= .002).

**TABLE 2:** Wechsler Adult Intelligence Scale (WAIS)‐IV scores distribution in the total study (*N* = 140), attention‐deficit/hyperactivity disorder (ADHD) (*n* = 77) and non‐ADHD (*n* = 63) groups.

	Total group, *N* = 140 M (SD)	ADHD group, *n* = 77 M (SD)	Non‐ADHD group, *n* = 63 M (SD)	95% CI of the difference			Group differences *p*‐value	Cohen's *d*
				Lower	Upper			
FSIQ	97.8 (13.0)	95.5 (12.8)	100.6 (12.8)	0.789		9.384	.021	.40
VCI	97.2 (14.3)	94.4 (13.6)	100.2 (14.6)	1.063		10.518	.017	.41
PRI	101.6 (15.2)	99.4 (15.0)	104.3 (15.2)	−.188		9.955	.059	.32
WMI	96.6 (14.1)	95.0 (13.8)	98.7 (14.3)	−1.022		8.381	.124	.26
PSI	98.5 (12.9)	97.4 (13.7)	99.8 (11.2)	−1.982		6.707	.284	.18

Abbreviations: FSIQ, full scale intelligence quotient; VCI, verbal comprehension index; PRI, perceptual reasoning index; WMI, working memory index; PSI, processing speed index.

An independent‐sample *t*‐test was used to compare the WAIS indices for the ADHD group and the non‐ADHD group (Table 3). Lower scores were obtained in the ADHD group for WAIS FSIQ (*t*(138) = 2.341 and *p = *.021) and VCI (*t*(138) = 2.422 and *p *= .017), but effect sizes were small.

**TABLE 3:** Comparison of Wechsler Adult Intelligence Scale (WAIS) indexes across attention‐deficit/hyperactivity disorder (ADHD) subtypes.

Measures	Non‐ADHD group, *n* = 63 M (SD)	ADHD‐combined type, *n* = 50 M (SD)	ADHD—inattentive, *n* = 27 M (SD)	Group differences *p*‐value
FSIQ	100.6 (12.8)	96.0 (13.7)	94.6 (11.2)	.063
VCI	100.2 (14.6)	95.0 (14.7)	93.4 (11.7)	.052
PRI	104.3 (15.2)	98.2 (16.0)	101.6 (12.8)	.108
WMI	98.7 (14.3)	97.0 (14.3)	91.3 (12.2)	.070
PSI	99.8 (11.2)	98.3 (14.2)	95.7 (14.3)	.399

FSIQ, full scale intelligence quotient; VCI, verbal comprehension index; PRI, perceptual reasoning index; WMI, working memory index; PSI, processing speed index.

When dividing the study group into non‐ADHD, ADHD‐combined subtype, and ADHD‐ inattentive subtype no significant difference was found between the three subgroups although the ADHD‐inattentive type come close to significantly lower results on FSIQ, VCI, and WMI.

We also compared WAIS results in the ADHD inattentive subgroup (*n* = 27) to the remaining collapsed study sub‐group including those with ADHD combined type and those with non‐ADHD (*n* = 113) and found that the ADHD inattentive subtype had significantly lower WMI (M = 91.3, SD = 12.2) versus (M = 97.9, SD = 14.4, *p *= .026; *t*(138) = 2.246, *p *= .026 (Table 4).

**TABLE 4:** Comparison of Wechsler Adult Intelligence Scale (WAIS) results subgroups with attention‐deficit/hyperactivity disorder (ADHD)‐inattentive type and non‐ADHD‐inattentive type.

Measures	ADHD—inattentive, *n* = 27 M (SD)	Non‐ADHD—inattentive, *n* = 113	*p*‐value
FSIQ	94.6 (11.2)	98.6 (13.3)	.156
VCI	93.4 (11.7)	97.9 (14.8)	.141
PRI	101.6 (12.8)	101.6 (165.8)	.991
WMI	91.3 (12.2)	97.9 (14.4)	.026*
PSI	95.7 (14.3)	99.1(12.6)	.222

FSIQ, full scale intelligence quotient; VCI, verbal comprehension index; PRI, perceptual reasoning index; WMI, working memory index; PSI, processing speed index ,*=p<0.05

### 3.2 Executive function tests

The total study group, regardless of ADHD diagnosis or not, performed significantly lower than the norms on the executive tests number‐letter switching (TMT, *t*(134) = −8.244, *p *< .001) and in Inhibition/ switching (CWIT, *t*(134) = −5.226, *p *< .001) (Table 5). However, no significant difference was found between the ADHD group and the non‐ADHD group on any executive function task even though there was a tendency for the ADHD group to perform worse than the non‐ADHD group at letter/category switching (verbal fluency). However, we found that the ADHD group had significantly lower scores in Letter sequencing (M = 6.7, SD = 4.0 vs. M = 8.1, SD = 2.9, *t*(135) = 2.296, *p *= .023) and color naming (M = 6.9, SD = 3.2 vs. M = 8.0, SD = 3.0, *t*(135) = 2.108, *p *= .037) compared to the non‐ADHD group. The total group, regardless of ADHD or not, scored below average (scale score <7) in the motor part in Trail making. When performing Bonferroni adjustment requiring a clinical value of *p *< .005, these significant differences disappeared.

**TABLE 5:** Standard scores on the trail making, word fluency, Color Word Interference Test, and twenty questions in the total study group (*N* = 136), the attention‐deficit/hyperactivity disorder (ADHD) group (*n* = 73) and the non‐ADHD group (*n *= 63).

	Total M (SD)	95% CI	ADHD group, M (SD	95% CI	Non‐ADHD group, M (SD	95% CI	*p*‐value
**Trail making**							
Visual scanning	9.1 (2.9)	(8.6, 9.6)	8.9 (2.9)	(8.3, 9.7)	9.3 (2.9)	(8.5, 10.0)	.387
Number sequencing	7.7 (3.2)	(7.1, 8.2)	7.4 (3.6)	(6.5, 8.1)	8.0 (2.7)	(7.3, 8.7)	.126
Letter sequencing	7.4 (3.6)	(6.8, 8.0)	6.7 (4.0)	(5.8, 7.7)	8.1 (3.0)	(7.4, 8.9)	.016*
Motor	6.1 (3.9)	(5.4, 6.7)	5.8 (3.7)	(4.9, 6.6)	6.4 (4.2)	(5.3, 7.5)	.241
Number/letter Switch	7.8 (3.3)	(7.2, 8.3)	7.2 (3.4)	(6.4, 8.1)	8.3 (3.0)	(9.5, 9.0)	.148
**Word fluency**							
Letter fluency, total	10.3 (3.4)	(9.7, 10.9)	10.0 (3.3)	(9.1, 10.8)	10.6 (3.6)	(9.8, 11.5)	.230
Category fluency, total	11.1 (3.7)	(10.5, 11.8)	10.6 (3.5)	(9.8, 11.5)	11.4 (3.8)	(10.5, 12.4)	.214
Letter/category, switch^a^	10.4 (3.4)	(10.8, 11.8)	9.9 (3.1)	(9.1, 10.7)	10.9 (3.5)	(10.1, 11.8)	.054
Letter/category, switch ^b^	11.3 (2.9)	(10.8, 11.8)	11.0 (2.8)	(10.3, 11.7)	11.7 (2.9)	(11.0, 12.4)	.156
**Color Word Interference Test**							
Color naming	7.4 (3.2)	(6.9, 8.0)	6.8 3.2)	(6.2, 7.7)	8.2 (2.9)	(7.2, 8.8)	.006**
Color reading	8.3 (3.0)	(7.8, 8.8)	8.0 (3.2)	(7.3, 8.8)	8.7 (2.8)	(7.8, 9.3)	.089
Inhibition	8.7 (3.6)	(8.1, 9.3)	8.2 (3.7)	(7.3, 9.1)	9.2 (3.4)	(8.3, 10.0)	.070
Inhibition/switching	8.5 (3.5)	(7.9, 9.1)	8.1 (3.9)	(7.1, 9.1)	8.8 (3.0)	(8.0, 9.5)	.597
Twenty questions							
Abstraction score	11.0 (2.8)	(10.5, 11.4)	10.9 (2.6)	(10.2, 11.6)	11.2 (2.8)	(10.5, 11.9)	.539

^a^ total correct responses;

^b^ total correct switching

*=p<0.05**=p<0.01

### 3.3 Logistic regression

We made a logistic regression to assess the impact of VCI, and the subtests of letter sequencing (Trail making) and color naming (Color Word Interference Test) on the dependent variable ADHD/non‐ADHD. These independent variables significantly differed between the ADHD and non‐ADHD group. We also added sex as an independent variable but excluded WMI since there was no significant difference between the ADHD/non‐ADHD group and excluded FSIQ since it is a composite score including VCI. The full model containing all predictors was statistically significant, *χ* ^2^ (4, *N* = 134) = 10.180, *p *= .038 indicating that the model was able to distinguish between the ADHD/non‐ADHD group. The model as a whole explained between 7.3% (Cox and Snell *R*‐squared) and 9.8% (Nagelkerke R squared of the variance) and correctly classified 60.4 % of the cases. As reported in Table 6, no predictors made a unique significant contribution to the model.

**TABLE 6:** Logistic regression predicting likelihood of attention‐deficit/hyperactivity disorder (ADHD) diagnosis.

							95% CI for Exp(B)	
	B	S.E.	Wald	*df*	Significance	Exp (B)	Lower	Upper
VCI	−.020	.014	2.123	1	.145	.980	.954	1.007
Color naming	−.071	.068	1.105	1	.293	.931	.816	1.063
Letter sequencing	−.062	.060	1.055	1	.304	.940	.836	1.058
Sex	−.580	.394	2.171	1	.141	.560	.259	1.211
Constant	3.500	1.351	6.715	1	.010	33.103		

Abbreviation: VCI, verbal comprehension index. Exp(B)=The predicted change in odds for a unit increase in the predictor

## 4 DISCUSSION

FSIQ in a young adult sample referred to an AOP showed FSIQ within the average range. The group with ADHD (including all subtypes) had significantly lower VCI (verbal comprehension) and FSIQ than the non‐ADHD group. Interestingly, working memory which is frequently reported to be significantly reduced in ADHD did not separate those with and without ADHD. The association between working memory and internalizing symptoms (anxiety and depression) in children and adolescents with or without ADHD was the focus in a study by Ferrin and Vance (2014). They found no differences in working memory between those with or without ADHD in the older age group when higher levels of internalizing symptoms occurred. This might partly explain why we found no differences in working memory in our psychiatric sample where about 14%–19% had an anxiety and/or affective disorder (Eberhard et al., 2022). Also, we did not find any differences in executive function results between the ADHD group and the non‐ADHD group, which in the literature are reported to be specifically negatively affected in young people with ADHD (Alderson et al., 2013; Wodka et al., 2007). This discrepancy might possibly be explained by adults with ADHD having lived with ADHD for decades could and might have developed compensating strategies, for example, to deal with impulsivity consequences (Canela et al. 2017).

It is of note that the ADHD group performed worse than the non‐ADHD group on tests of letter sequencing (TMT), where the participant connects letters in order, and in color naming (Color Word Interference Test), where the participant is asked to say the names of colors presented on a page, both tests as quickly as he/she can. These two tests are not primary tests of executive function but involve aspects of executive function such as processing speed. Difficulties on tasks requiring fast processing of colored stimuli in ADHD have previously been reported and suggested to reflect subtle impairments in the perceptual encoding stage of stimulus color, which might be associated with hypodopaminergic functioning (Tannock et al., 2006). It might also be explained by the effect of psychiatric comorbidity which has shown a negative effect on both executive function and other cognitive functions (Eberhard et al., 2022). Surprisingly, we also found that the total group, regardless of existence of ADHD or not, performed below average in the motor part of Trail making. Motor speed is often neglected in neuropsychological assessment in favor of executive functions despite the fact that signs of motor dysfunction have been evidenced across a range of psychiatric disorders (Correa‐Ghisays et al., 2017; Gillberg et al., 1981; Mittal et al., 2017). Our study suggests that slow motor speed occurs generally in psychiatric conditions. What impact this has on adaptive functioning is however not clear.

We found no significant result differences on the D‐KEFS test, which has been specifically designed to measure executive function. We only found statistically significant differences between the ADHD and the non‐ADHD subgroup in the domains of letter sequencing and color naming, but after Bonferroni adjustments these differences disappeared. These are not primarily tests for executive functioning but rather tests that are used as control tests in the test battery. When we entered the results from these and the results from the VCI from WAIS into a logistic regression model, we found that the model explained only between 7% and 10% of the variance. This means that there are many other “specific” factors that are unaccounted for in relation to ADHD diagnosis or not. This also means that executive function tests may not be useful when it comes to discriminate between adults with and without ADHD in AOP. The result from our study supports the strategic research plan of the National Institute of Mental health (www.nimh.nih.gov/strategicplan) that proposes the need for classifying mental disorders based on dimension of measures relating to behavior and neurobiology.

However, these tests might be useful in clinical practice as a therapeutic tool to highlight some deficits (and even possible relative strengths) in executive function to certain individual patients. As a pure diagnostic tool, our results show that it is difficult or even misleading to use them. However, we do not want to underestimate the importance of overall intellectual assessment, since this provides important information in relation to the differential diagnostic process of intellectual disability and to identify borderline intellectual functioning. Knowing the overall cognitive functioning will help to clarify if general expectations regarding academic, adaptive, or social performances have been pitched too high (or in a few cases, too low). We found a significant difference between the two groups regarding FSIQ and VCI. Rather surprisingly, we found no differences in working memory results across the two subgroups ADHD/non‐ADHD albeit, lower working memory results were found in the ADHD inattentive type compared to the remaining participants (ADHD combined type and non‐ADHD). This indicates that the different subtypes have different origins and may reflect that ADHD subtypes should be assessed in different ways.

### 4.1 Clinical implications

The standard procedure for assessing neurodevelopmental disorders in many clinics include a large battery of neuropsychological tests. The result of our study suggests that, other than with regard to overall cognitive performance profile, the clinical implication of such testing is, at best, small, particularly if the purpose is to “rule out” an ADHD diagnosis. However, the use of tests such as the WAIS is recommended in the differential diagnostic process, particularly with a view to finding cases with “comorbid” borderline intellectual function or intellectual disability.

### 4.2 Limitation

There are three major limitations that need to be considered. About 20% of the total original study group did not take part in the cognitive assessment. Also, even though the study group targeted and included all attendees at an AOP in Stockholm, Sweden, the results cannot be generalized to adult age groups of psychiatric patients with or without ADHD. Finally, the sample size is a limitation, emphasizing the need for future larger studies

## AUTHOR CONTRIBUTIONS

**D. Eberhard**: Investigation; writing—original draft; conceptualization; formal analysis. **C. Gillberg**: Supervision; conceptualization; writing—review and editing. **E. Billstedt**: Conceptualization; supervision; writing—review and editing; formal analysis.

## CONFLICT OF INTEREST STATEMENT

The authors declare no conflicts of interest.

### PEER REVIEW

The peer review history for this article is available at https://publons.com/publon/10.1002/brb3.3626

## Data Availability

Research data are not shared.
